# Evaluation of Tilt-correction of Anteversion on Anteroposterior Pelvic Radiographs in Total Hip Arthroplasty

**DOI:** 10.7759/cureus.2647

**Published:** 2018-05-18

**Authors:** Jeffrey M Muir, John Vincent, Joseph Schipper, Meinusha Govindarajan, Wayne G Paprosky

**Affiliations:** 1 Clinical Research, Intellijoint Surgical; 2 Faculty of Applied Health Sciences, School of Public Health and Health Systems, University of Waterloo; 3 Research & Development, Intellijoint Surgical; 4 Surgery, Central Dupage Hospital

**Keywords:** total hip arthroplasty, acetabular cup position, pelvic tilt, anteversion, tilt-correction

## Abstract

Despite inaccuracies due to artifact and variations in patient positioning, anteroposterior (AP) radiographs remain the clinical standard for post-operative evaluation of component placement following total hip arthroplasty (THA). However, cup position, specifically anteversion, can be significantly affected by variations in patient positioning on an X-ray. A major cause of such artifact is unaccounted for pelvic tilt. Several methods for correcting the effects of pelvic tilt on radiographic anteversion have been proposed, with varying degrees of accuracy. The purpose of this study was to evaluate the accuracy and reliability of a commonly referenced method for correcting acetabular cup anteversion in a cohort undergoing total hip arthroplasty and determine its appropriateness for use in this population of patients. Radiographs from patients who underwent primary or revision hip arthroplasty between February 2016 and February 2017 were retrospectively reviewed. Corrected anteversion was calculated by measuring the vertical distance between the symphysis pubis and the sacrococcygeal joint, per the method outlined by Tannast et al. This symphococcygeal distance was then applied to Tannast’s nomograms to calculate the magnitude of pelvic tilt. Corrected and uncorrected anteversion values were compared to anteversion values collected intraoperatively using an imageless computer-assisted navigation device. A total of 71 cases were initially eligible for inclusion in the study. The correction method could not be applied in 44% (31/71) of the cases, chiefly due to difficulties in visualizing the required landmarks. In cases where it could be applied, corrected values correlated very poorly with navigation measurements (r = -0.07). Mean corrected anteversion (36.9°, SD: 7.4°) differed from uncorrected anteversion (25.2°, SD: 7.6°) by an average of 13.5° (p<0.001). Mean navigated anteversion (27.4°, SD: 5.7°) differed from corrected values by an average of 10.8° (p=0.16). The evaluated correction method could not be consistently applied to radiographs and did not reliably correct anteversion due to pelvic tilt in this population of patients undergoing hip arthroplasty. This correction method does not appear to be appropriate for use in this patient population.

## Introduction

Proper positioning of the acetabular cup component in total hip arthroplasty (THA) is vital; as inaccurate placement can lead to accelerated component wear, component loosening, reduced functional capacity, and an increased risk of impingement or dislocation [1-3]. Accurate radiographic assessment of cup orientation is therefore essential when evaluating the outcome of THA surgery.

Despite their known susceptibility to distortion and artifact, radiographs remain the standard of care for imaging, due to their low radiation exposure and easy accessibility [4]. Consistent positioning of patients during radiographs remains the chief challenge to imaging accuracy, as changes in leg adduction/abduction and pelvic tilt can dramatically alter the accuracy of measurements obtained from radiographs [5]. As such, plain radiographs may be inadequate for evaluating cup position if variations such as pelvic orientation are not taken into account [6].

Pelvic tilt has been a subject of much discussion regarding its effect on radiographic accuracy, as variations in pelvic tilt alter the projection of the pelvis onto the two-dimensional radiograph, introducing error to the measurement of acetabular orientation [7-8]. This error is generally considered negligible for inclination [9], but anteversion is highly susceptible to changes in pelvic tilt since its measurement is based solely on the shape of the ellipsis created by the cup surface on the radiograph [10]. To account for this projection error, several methods to correct pelvic tilt on radiographs have been presented in the literature [7,11], but have yet to achieve general acceptance for clinical use. In a recent study, Tannast et al. [7] evaluated several proposed measurements and found that the vertical distance from the pubic symphysis to the sacrococcygeal joint (symphococcygeal distance, (SCD)) correlated well with pelvic tilt. Using this method, the authors developed a nomogram for easy calculation of “tilt-corrected anteversion”. However, the broad application of their preferred method may be difficult, as their radiographic methods were highly standardized and deviate from standard clinical procedure. Furthermore, as they used a cohort of young patients, the validity of their correction method in an older population undergoing THA is in question.

The objective of this study was to evaluate the accuracy and reliability of the SCD method to correct radiographic anteversion on standard post-operative radiographs in a cohort of patients undergoing THA. We compared radiographic measurements before and after correction with values calculated from an imageless computer-assisted navigation system.

## Materials and methods

Study design

This investigation was a retrospective, single-centre, clinical study of patients undergoing primary or revision total hip arthroplasty. All included patients provided informed consent. Ethics approval was received prior to data collection.

Patients

Patients were eligible for inclusion if they underwent a primary or revision THA performed by the senior author (WGP) between February 2016 and February 2017. Specific inclusion criteria included THA using the Intellijoint HIP® navigation system and ability to obtain two-week postoperative anteroposterior (AP) pelvic radiographs. Patients were excluded from analysis if the navigation tool was removed for any reason prior to the recording of necessary measurements, or if AP pelvic radiographs or navigation values were unable to be retrieved post-operatively.

Radiographic data

**Figure 1:**
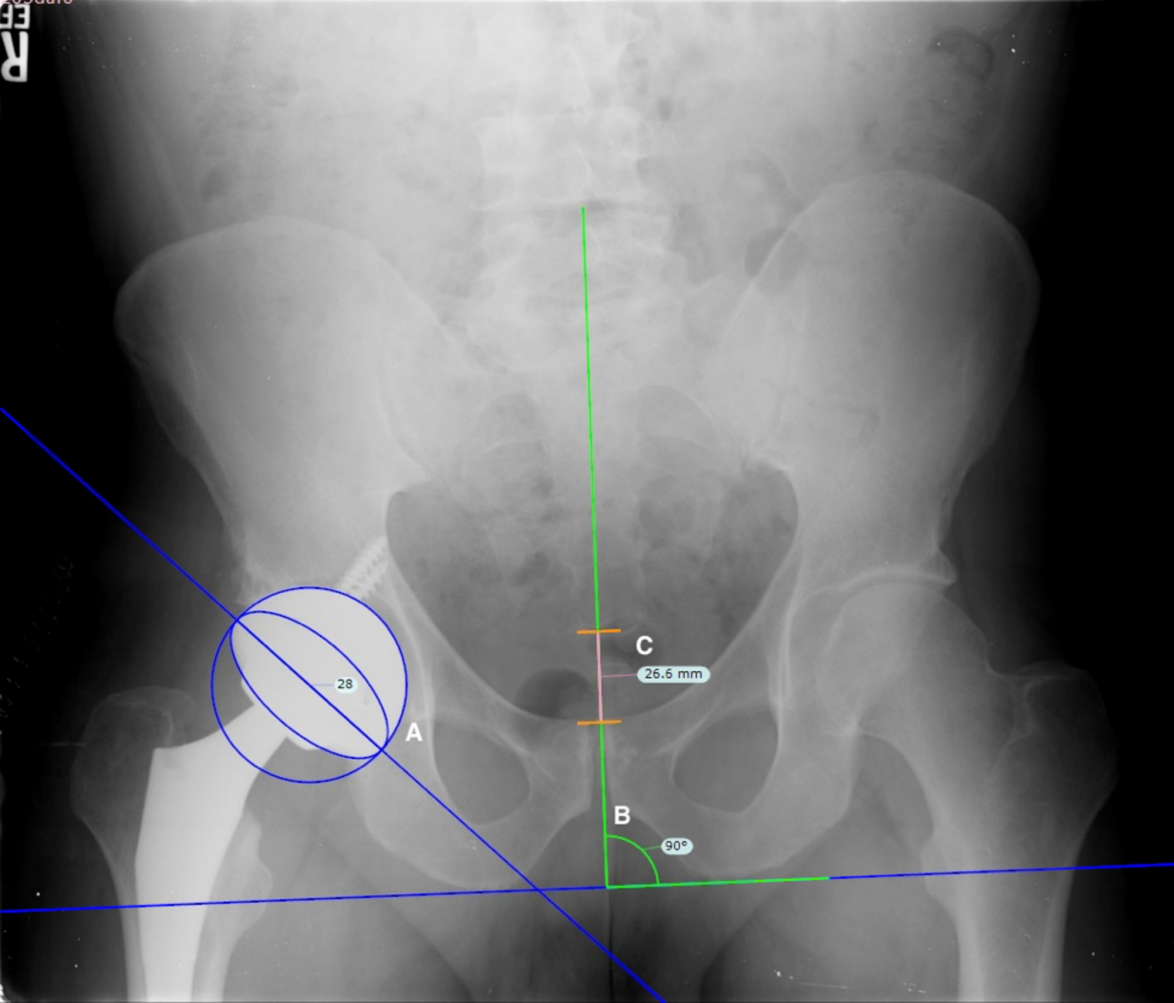
Tannast method Radiographic analysis illustrating measured cup anteversion (A), the identification of “vertical” relative to the trans-ischial line (B), and measured symphococcygeal distance from the pubic symphysis to the sacrococcygeal joint (C).

Radiographic analysis was performed on standing, two-week post-operative AP radiographs using TraumaCad (version 2.5, Brainlabs, Chicago, IL). Radiographs were scaled using the known diameter of the implanted femoral head. Pelvic tilt (deviation of the anterior pelvic plane (APP) from the coronal reference plane [11-12]) was estimated using the method described by Tannast et al. [7], where the vertical distance between the upper edge of the pubic symphysis to the centre of the sacro-coccygeal joint is measured. In this context, vertical was defined as a line perpendicular to the trans-ischial line, to account for any pelvic obliquity (Figure 1).

Anteversion was measured using the trans-ischial line method. All radiographic measurements were made in triplicate and the results averaged to provide a final value. After completing radiographic measurements, the gender-specific nomograms created by Tannast et al. were used to find the tilt-corrected anteversion. The nomograms correlate the SCD with the measured (uncorrected) anteversion to determine the tilt-corrected value. The measurements and application of the nomogram were completed by a single researcher (JV), who was blinded to navigation values.

Navigation data

Intraoperative measurements of anteversion were measured using a computer-assisted navigation device (Intellijoint HIP® navigation system, Intellijoint Surgical, Waterloo, ON). The indications and details on device use and workflow are described elsewhere [13-14]. In brief, the device consists of a camera, mounted to the operative iliac crest via two surgical pins, and a tracker, which can be magnetically fixed to a platform mounted to the greater trochanter or to surgical tools (e.g., impactor). During registration, patient position and pelvic orientation are registered relative to the patient's frontal plane. As the camera remains fixed to the patient during surgery and intraoperative measurements are calculated relative to this initial registration orientation, the resulting anteversion measurements represent the true in situ anteversion, not one distorted by imaging or intraoperative changes in pelvic position or orientation.

Study outcomes and statistical analysis

Acetabular cup anteversion measured from post-operative radiographs was compared with anteversion corrected using the nomograms provided by Tannast et al. [7] and anteversion measured intraoperatively by the navigation device.

Alpha was set a priori to p<0.05 for all statistical comparisons between the radiographic and navigation data. Intra-observer reliability was assessed using the intra-class correlation coefficient. Mean values are expressed as mean (standard deviation) and compared using the mean differences and Student’s t-test or single-factor ANOVA as appropriate. Correlations were evaluated using Pearson’s r.

## Results

Patient population

Seventy-seven eligible cases were completed during the study period. One patient was excluded due to the presence of a pelvic fracture on post-operative radiographs, and five patients were excluded due to extremely poor quality post-operative radiographs, from which accurate measurements were not possible. A final sample of 71 cases for which navigation data and uncorrected radiographs were available was included for analysis and comprised the baseline group.

**Figure 2:**
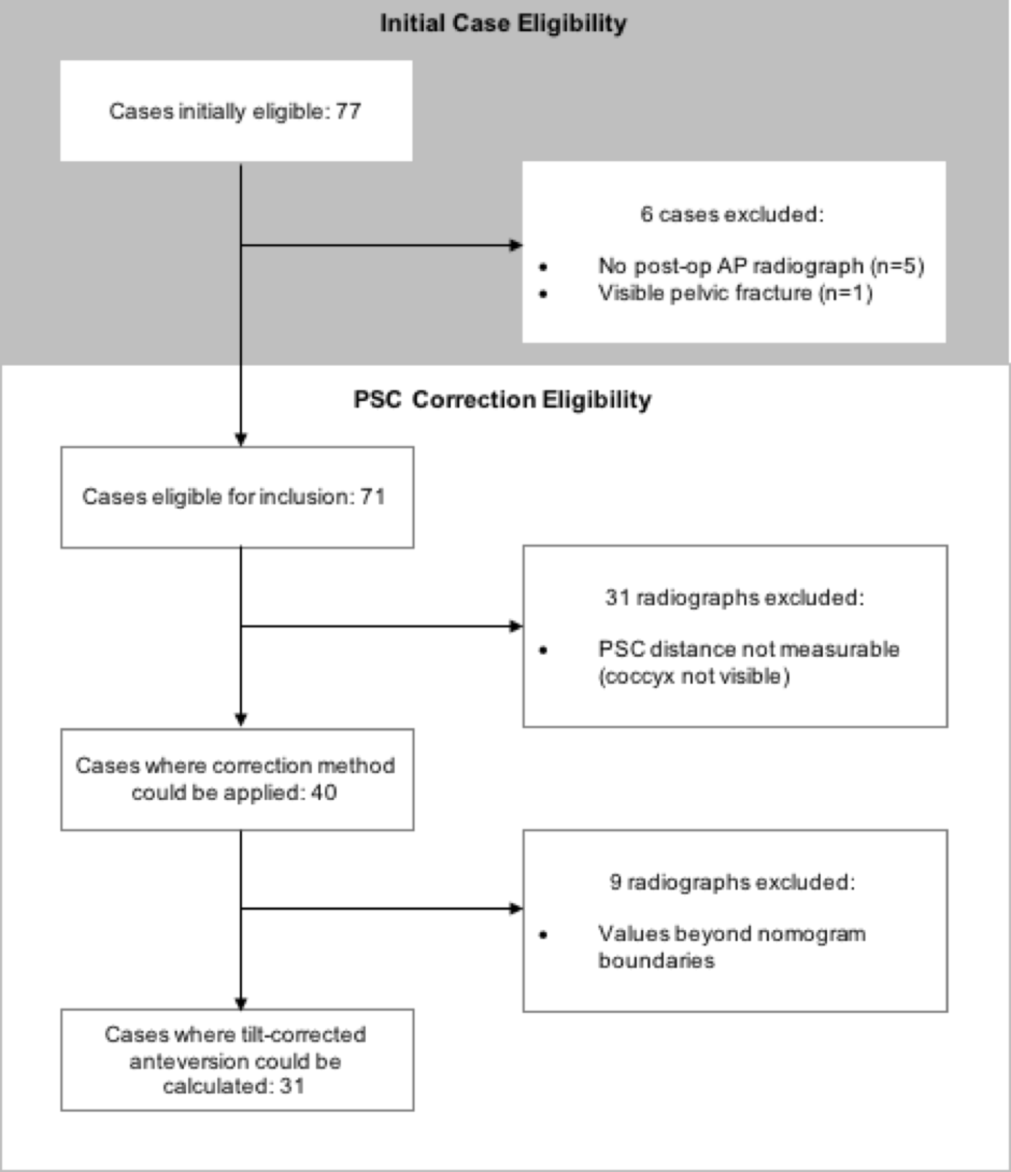
Case flow-through Case flow-through and data collection eligibility.

In this baseline group, the sacrococcygeal joint was not visible in 44% (31/71) of radiographs. In the remaining 40 radiographs, the anteversion and/or SCD measurements calculated from the image were beyond the scale provided by the nomograms in 23% (9/40). As a result, the correction method was able to be successfully applied to only 44% (31/71) of radiographs from the initially eligible cases (correction-eligible group) (Figure 2).

No statistically significant differences were noted between gender distribution, operative side, or age in the baseline and correction-eligible groups (Table 1).

**Table 1:** Demographic statistics

	Baseline cohort (n=71)	Correction-eligible cohort (n=40)	p-value*
Gender			0.39**
Male, n (%)	38 (54)	18 (45)	
Female, n (%)	33 (46)	22 (55)	
Operative side			0.84**
Right, n (%)	27 (38)	16 (40)	
Left, n (%)	44 (62)	24 (60)	
Age, mean (years)			
Male and female	63.3	63.1	0.88***
Male only	62.2	60.0	0.35***
Female only	64.7	65.8	0.59***

Intra-observer reliability

Intra-observer reliability between the three rounds of measurement was excellent for both anteversion measured from radiographs (intraclass correlation = 0.99, SD: 0.51°) and the symphococcygeal distance itself (intraclass correlation = 0.99, SD: 0.25 mm).

Effect of correction on anteversion values

Anteversion in the baseline and correction-eligible cohorts did not differ significantly for either radiographic or navigation measurements. Uncorrected radiographic measurements of anteversion were 26.3° (SD: 6.8°) in the baseline cohort and 25.2° (SD: 7.6°) in the correction-eligible cohort (p=0.46), while navigation values were 27.1° (SD: 5.3°) and 27.4° (SD: 5.7°), respectively (p=0.79).

**Figure 3:**
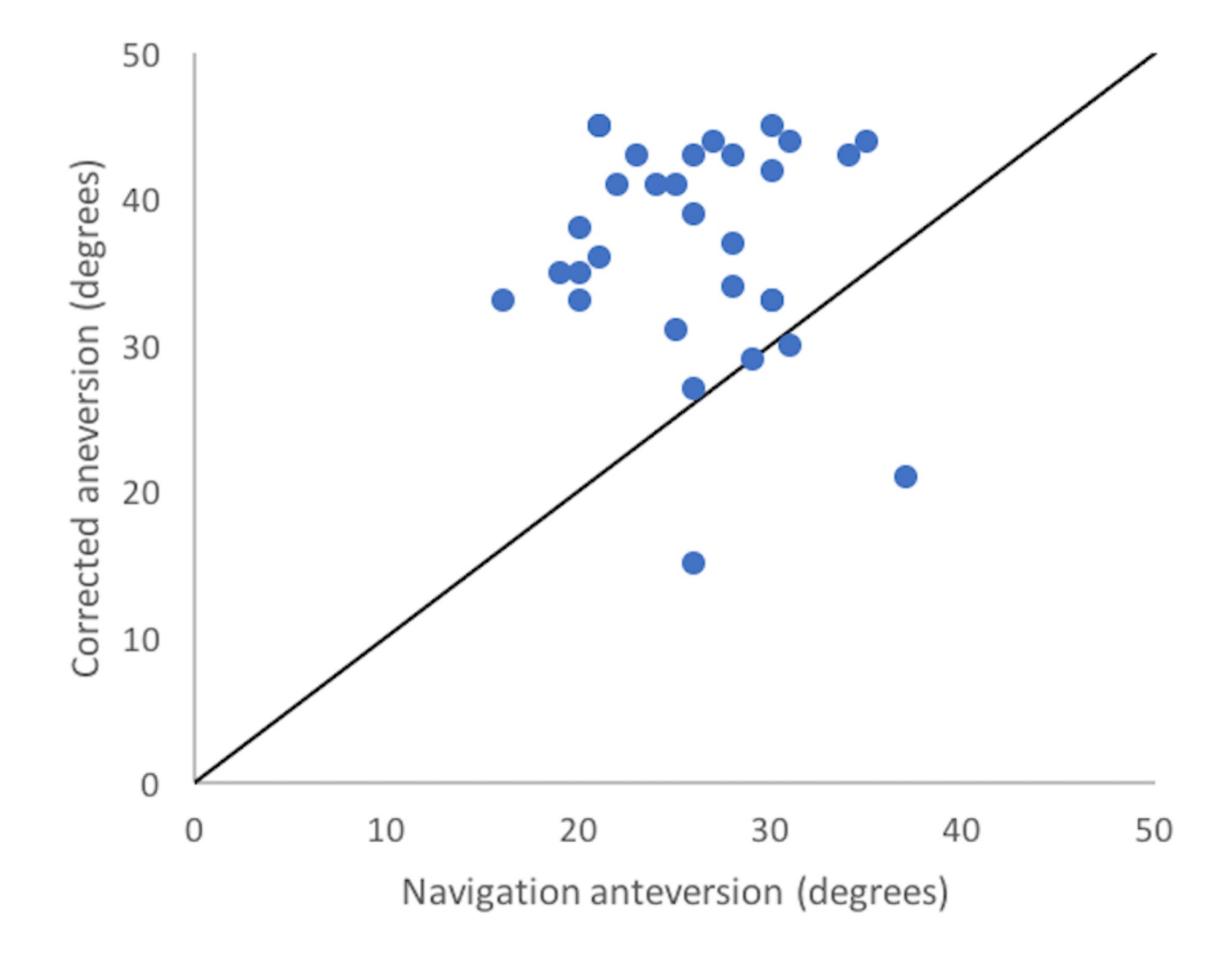
Corrected vs. navigated Scatterplot showing the comparison between corrected anteversion values with navigation values.

**Figure 4:**
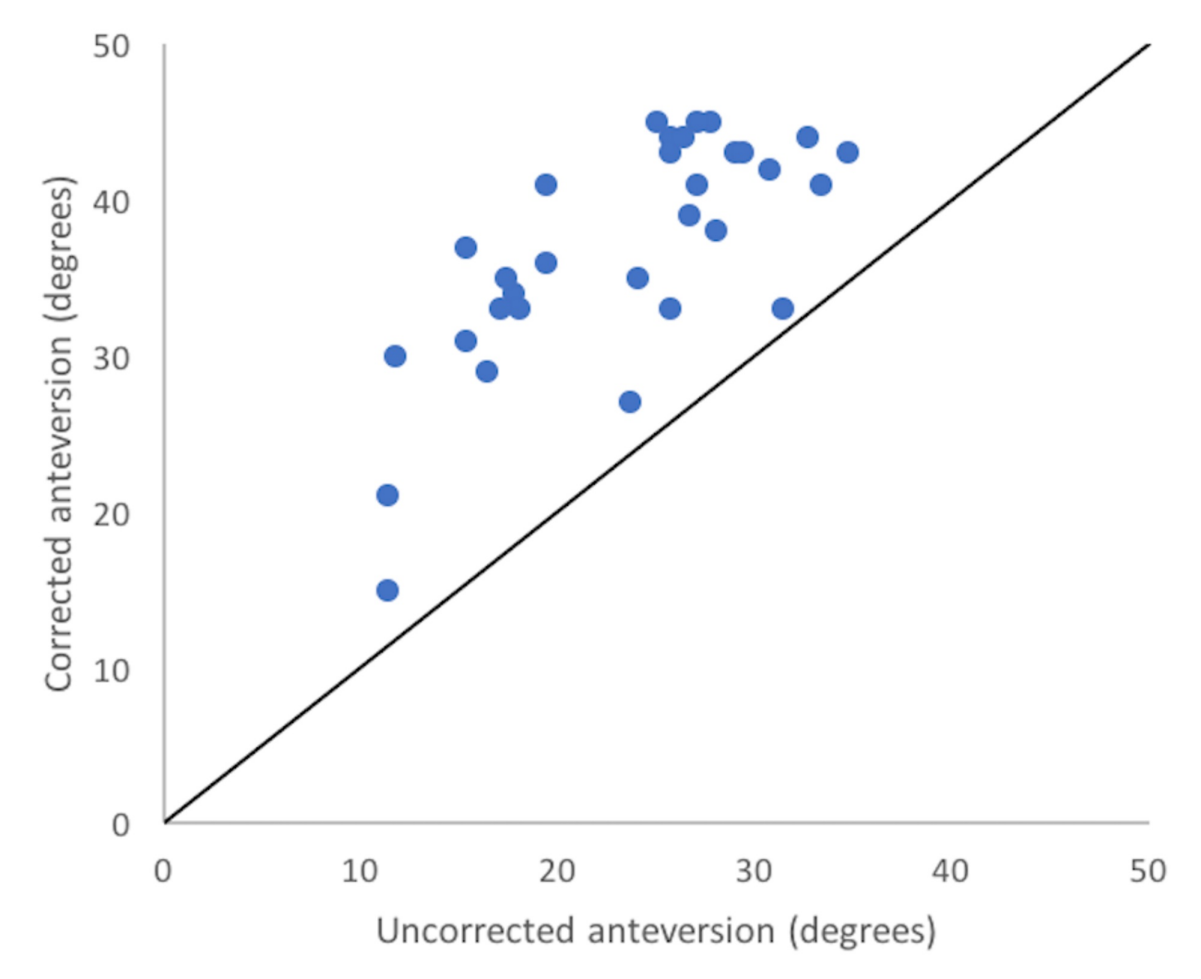
Corrected vs. uncorrected Scatterplot showing the comparison between corrected anteversion values with uncorrected radiographic values.

In the correction-eligible cohort (n=40), uncorrected, corrected and navigation values differed significantly (F=27.38, p<0.0001). Corrected anteversion (mean: 36.9°, SD: 7.4°) differed from uncorrected anteversion (mean: 25.2°, SD: 7.6°) by an average of 13.5° (p<0.001) and from navigated anteversion (27.4°, SD: 5.7°) by an average of 10.8° (p=0.16). Navigation values did not differ significantly from uncorrected radiographic values (27.4° (SD: 5.7°) vs. 25.2° (SD: 7.6°), p=0.16). Corrected anteversion correlated very poorly with navigation values (r = -0.07) (Figure 3) although moderately well with uncorrected values (r = 0.73) (Figure 4). 

**Figure 5:**
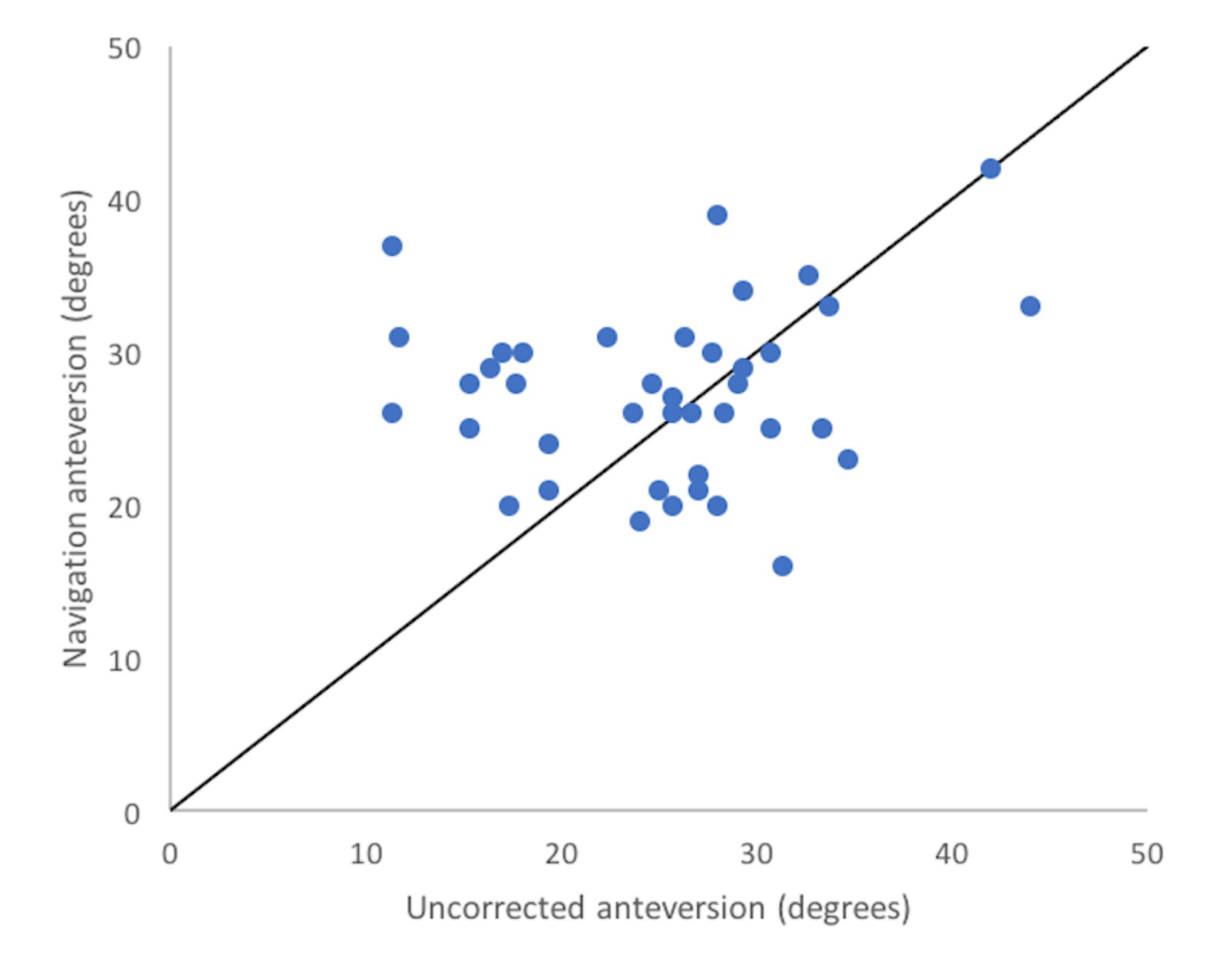
Navigated vs. uncorrected Scatterplot illustrating the correlation between uncorrected and navigated anteversion values, showing uniform correlation between the two measurements.

Uncorrected anteversion correlated poorly with navigation values (r = 0.15) but differed by an average of only -2.2° (SD: 8.7°) and demonstrated a more uniform distribution when compared with the uniform increase in anteversion values resulting from the correction method (Figure 5). 

## Discussion

Accurate evaluation of acetabular cup position after THA is crucial for assessing treatment success, as malpositioning increases the risk of numerous post-operative sequelae that may necessitate further intervention [9,15-16]. While radiographs are the standard post-operative imaging modality, the inaccuracy of radiographic methods for determining cup anteversion and their inability to correct for positional errors such as pelvic tilt are well-documented [5-6,8,11,17]. Several methods for correcting for pelvic tilt have been suggested, with limited success. Our evaluation of one common correction method [7] found that this method did not provide a consistently valid correction for radiographic anteversion.

We noted several deficits when applying the SCD correction method that suggest that it may not provide a suitably reliable method for tilt correction. Primarily, the inability to reliably identify the required landmarks on radiographs presented a significant obstacle. We were unable to identify the coccyx in 44% of cases, immediately limiting our ability to broadly apply the correction method. In the group of cases where we were able to apply the correction, the measured values fell outside of the limits of the nomograms in a substantial portion of cases (23%). Finally, in those cases where a corrected anteversion value could be calculated, there were significant differences between the corrected anteversion values and those calculated by the navigation device. No strong correlations were noted between corrected, uncorrected and navigation anteversion values. In fact, navigated values were more uniformly correlated with uncorrected values than corrected values. Corrected values were observed to be increased in all cases when compared with both navigated and uncorrected values. This trend towards increasing anteversion has important implications, as it could lead to overestimation of intraoperative anteversion, resulting in lower true anteversion orientations, a risk factor for posterior dislocation [18]. As such, careful interpretation of corrected anteversion is required to minimize the potential exposure of patients to increased risk of dislocation.

There were important methodological differences between our study and that of Tannast et al. that may help to explain the observed discrepancies. Firstly, Tannast et al. employed highly standardized radiographic techniques with a controlled source-to-image distance of 1.2m, patients in supine position, and the central beam aimed at the midpoint between the pubic symphysis and centre of the trans-ASIS line. This highly regimented approach is in contrast with standard post-operative AP radiographs, which are taken in either supine or standing position, with the central beam aimed at the pubic symphysis, and commonly with small variations in source-to-image distance [19]. Second, differences in population demographics (mean age: 31.7 yrs vs. 63.1 yrs) and indication (femora-acetabular impingement or developmental dysplasia vs. THA) and the associated anatomical differences that may account for the discrepancies and underscore the questionable validity of this correction method for THA patients. Finally, methods for measuring the SCD may be inconsistent between studies. Tannast et al. did not clarify whether their vertical distance was relative to a pelvic reference (such as the trans-ischial line) or relative to the plane of the X-ray, and failed to record if or how they scaled their radiographs. While most templating software assumes a magnification factor of 115% or 120% [20-21], true magnification can range from 97% to 127% [20-24]. We scaled radiographs using the known head size of the femoral implant, a reliable method that has been used elsewhere [21]. Since Tannast et al. used a highly standardized radiographic method, magnification should not impact their internal validity, but may explain the low compatibility of the correction method with the radiographs in the present study.

The retrospective nature of our study could be associated with limitations related to the consistency of the radiographic technique used, resulting in potential discrepancies between pre- and post-operative patient positioning. As well, comparing post-operative imaging with intraoperative measurements from navigation may represent a source of error, given that navigation measurements are intraoperative, while radiographs are post-operative. However, navigation in general [25-27] and the system utilized in this study specifically [13,28-29] have demonstrated excellent accuracy when compared with post-operative radiographs, with the Intellijoint system associated with an error of less than 3° when compared with radiographs [29].

## Conclusions

Our evaluation of a pelvic tilt correction method based on the symphococcygeal distance was associated with difficulties in identifying the required landmarks on X-ray and with significant discrepancies when compared with intraoperative measurements. While methodological differences may account for these discrepancies, the low reliability of the proposed method suggests it is not a viable option to correct for pelvic tilt in THA imaging. Further research is required to determine a more accurate method of tilt correction on radiographs.
